# Association of genetically predicted testosterone with thromboembolism, heart failure, and myocardial infarction: mendelian randomisation study in UK Biobank

**DOI:** 10.1136/bmj.l476

**Published:** 2019-03-06

**Authors:** Shan Luo, Shiu Lun Au Yeung, Jie V Zhao, Stephen Burgess, C Mary Schooling

**Affiliations:** 1 School of Public Health, University of Hong Kong, Hong Kong SAR, China; 2 Cardiovascular Epidemiology Unit, Department of Public Health and Primary Care, University of Cambridge, Cambridge, UK; 3 MRC Biostatistics Unit, University of Cambridge, Cambridge, UK; 4 School of Public Health and Health Policy, City University of New York, 55 West 125th Street, New York, NY 10027, USA

## Abstract

**Objective:**

To determine whether endogenous testosterone has a causal role in thromboembolism, heart failure, and myocardial infarction.

**Design:**

Two sample mendelian randomisation study using genetic variants as instrumental variables, randomly allocated at conception, to infer causality as additional randomised evidence.

**Setting:**

Reduction by Dutasteride of Prostate Cancer Events (REDUCE) randomised controlled trial, UK Biobank, and CARDIoGRAMplusC4D 1000 Genomes based genome wide association study.

**Participants:**

3225 men of European ancestry aged 50-75 in REDUCE; 392 038 white British men and women aged 40-69 from the UK Biobank; and 171 875 participants of about 77% European descent, from CARDIoGRAMplusC4D 1000 Genomes based study for validation.

**Main outcome measures:**

Thromboembolism, heart failure, and myocardial infarction based on self reports, hospital episodes, and death.

**Results:**

Of the UK Biobank participants, 13 691 had thromboembolism (6208 men, 7483 women), 1688 had heart failure (1186, 502), and 12 882 had myocardial infarction (10 136, 2746). In men, endogenous testosterone genetically predicted by variants in the *JMJD1C* gene region was positively associated with thromboembolism (odds ratio per unit increase in log transformed testosterone (nmol/L) 2.09, 95% confidence interval 1.27 to 3.46) and heart failure (7.81, 2.56 to 23.8), but not myocardial infarction (1.17, 0.78 to 1.75). Associations were less obvious in women. In the validation study, genetically predicted testosterone (based on *JMJD1C* gene region variants) was positively associated with myocardial infarction (1.37, 1.03 to 1.82). No excess heterogeneity was observed among genetic variants in their associations with the outcomes. However, testosterone genetically predicted by potentially pleiotropic variants in the *SHBG* gene region had no association with the outcomes.

**Conclusions:**

Endogenous testosterone was positively associated with thromboembolism, heart failure, and myocardial infarction in men. Rates of these conditions are higher in men than women. Endogenous testosterone can be controlled with existing treatments and could be a modifiable risk factor for thromboembolism and heart failure.

## Introduction

Testosterone sales increased 12-fold globally from 2000 to 2011,^1^ with noticeable increases in North America.^1^ In the United States, older men have probably been targeted in addition to men requiring medically indicated treatment for low testosterone levels.^1^ ^2^ Testosterone replacement therapy (TRT) prescriptions in the US decreased by 50% between 2013 and 2016,^3^ but remain well above the levels needed to treat pathological hypogonadism.^4^ Since the 1970s, use of anabolic steroids has spread from athletes to the general population,^5^ with a lifetime prevalence rate of 6.4% for men.^6^

Observational studies of the association of measured endogenous testosterone with overall and specific cardiovascular diseases, including thromboembolism,^7^ ^8^ ^9^ myocardial infarction,^10^ and heart failure,^11^ are difficult to interpret.^12^ ^13^ ^14^ ^15^ These studies are inherently open to confounding by obesity and ill health, which could reduce testosterone^16^ ^17^ and are well established causes of cardiovascular diseases.^18^ ^19^ Therefore, it is unclear from observational studies whether testosterone has a role or is an indicator of poor general health,^17^ particularly because testosterone might be affected by some cardioprotective treatments.^20^ Pharmacoepidemiology studies of drugs are open to subtle time related biases, such as immortal time bias,^21^ which are not always taken into consideration in studies of TRT.^22^ Studies of TRT using self comparisons to avoid confounding and time related biases have suggested that TRT could increase myocardial infarction^23^ and possibly cardiovascular events.^24^ Randomised placebo controlled trials of TRT are limited in size and scale. Therefore, systematic reviews and meta-analyses of randomised controlled trials are usually too small to be definitive overall or for any specific types of cardiovascular disease,^25^ although adverse effects of TRT on thromboembolism have been found.^26^ Recent Endocrine Society clinical practice guidelines recommend against TRT in men with stroke, myocardial infarction, or thrombophilia.^27^ To our knowledge, the new TRAVERSE trial (study to evaluate the effect of TRT on the incidence of major adverse cardiovascular events and efficacy measures in men with hypogonadism; clinicaltrials.gov, NCT03518034) is the first TRT trial with adequate power to assess cardiovascular events. The trial is designed to evaluate major adverse cardiac events (non-fatal myocardial infarction, non-fatal stroke, or death due to cardiovascular causes) in 6000 men over five years; it is unlikely to provide definitive evidence about specific cardiovascular diseases and will take several years to complete.

When the role of TRT is hotly debated but experimental evidence is limited, mendelian randomisation using genetic variants as instrumental variables can support causal inferences about the effects of modifiable risk factors.^28^ ^29^ ^30^ Mendelian randomisation is less susceptible to confounding than traditional observational studies because genetic variants are randomly allocated at conception. Therefore, mendelian randomisation is at the interface of experimental and observational studies,^30^ and can be used to obtain evidence in support of a potential causal effect or of potential targets of interventions.^30^ However, mendelian randomisation studies give the effects of lifetime exposures and so the numerical effect estimates provide a guide rather than the exact level of an intervention required.^30^ A previous adequately powered mendelian randomisation study found preliminary evidence that endogenous testosterone is detrimental for ischaemic heart disease and ischaemic stroke, especially in men.^31^ To clarify the role of testosterone in other types of cardiovascular disease, which might have different causes,^32^ we assessed the effect of endogenous testosterone on additional cardiovascular conditions in the UK Biobank participants: thromboembolism because of evidence from randomised controlled trials and specific warnings by the US Food and Drug Administration and Health Canada,^33^ ^34^ ^35^ heart failure because it can be a sequela of heart attack; and myocardial infarction for specificity. We validated our findings, when possible, using publicly available consortiums. We also considered whether the associations of genetic predictors of endogenous testosterone with the cardiovascular diseases studied varied by sex because men have higher levels of endogenous testosterone than women.

## Methods

We conducted a two sample mendelian randomisation study. Instrumental variable analysis with genetic instruments assumes the genetic variants that predict the exposure are not associated with the confounders of the exposure and outcome relation; they are only associated with the outcome through the exposure (exclusion restriction assumption). We obtained genetic predictors of serum testosterone from the largest available genome wide association study and we used the UK Biobank to assess the associations of these predictors with thromboembolism, heart failure, and myocardial infarction.^36^ We validated our findings using the CARDIoGRAMplusC4D 1000 Genomes based genome wide association study.^37^

### Participants

#### Genetic predictors of endogenous testosterone

We obtained genetic predictors of endogenous testosterone from a genome wide association study of log transformed serum testosterone conducted in 3225 men of European ancestry aged 50-75. These men consented to participate in genetic studies in the Reduction by Dutasteride of Prostate Cancer Events (REDUCE) randomised controlled trial.^38^ This trial excluded men who had used any form of TRT within the past 12 months. Baseline characteristics of participants who did and did not consent to genetic studies were similar. Serum testosterone was measured by high turbulent flow liquid chromatography coupled with tandem mass spectrometry, with sensitivity and specificity that met the acceptance criteria.^39^ Mean testosterone was 15.55 nmol/L with a standard deviation of 6.12.^38^ The Illumina HumanOmniExpress BeadChip (San Diego, CA, USA)****was used****for genotyping and imputation to a reference set combining 1000 Genomes pilot 1 and HapMap3. Variants with minor allele frequency less than 0.01, call rate less than 95%, or deviating from Hardy-Weinberg equilibrium (P<0.001) were excluded.^38^

Variants in two autosomal gene regions (*JMJD1C* at 10q21 and *SHBG* at 17p13) were identified as associated with serum testosterone at genome wide significance.^38^ The lead variants in these gene regions explain about 1.1% (*JMJD1C*) and 1.4% (*SHBG*) of the variance of serum testosterone.^38^ As in a previous study, we started with full lists of fine mapped variants at *JMJD1C* (661) and *SHBG* (325). The variants were checked for validity as instrumental variables using individual level data from the UK Biobank, according to the following exclusion criteria: imputation information score less than 0.6; departure from the Hardy-Weinberg equilibrium at Bonferroni corrected significance; or violation of the mendelian randomisation assumption that the genetic variant should be unrelated to factors potentially confounding any association with the outcomes, including baseline age, body mass index, socioeconomic status (Townsend index and educational level), and lifestyle factors (smoking and drinking) at Bonferroni corrected significance. Of the remaining genetic variants, we selected those with the lowest P value having a pairwise squared correlation (r^2^) less than 0.4. Population specific correlations between variants were estimated from LDlink^40^ ****using the 1000 Genomes Project (phase 3). We aligned the effect allele of each genetic variant on the serum testosterone increasing allele.

#### Genetic associations with thromboembolism, heart failure, and myocardial infarction

The UK Biobank recruited around 500 000 participants aged 40-69 in 2006-10. Participants provided biological samples, completed questionnaires, underwent assessments, and had nurse led interviews. Follow-up using record linkage to all health service encounters and mortality data is ongoing.^41^ We used data from the UK Biobank provided by the update on 11 April 2018. Genotyping was undertaken with two very similar arrays from Affymetrix (Santa Clara, California): the UK BiLEVE array and the UK Biobank Axiom array. Genotype imputation to a reference set combining the UK10K haplotype and the Haplotype Reference Consortium reference panels was performed.^42^ To reduce confounding by population stratification, we restricted our analysis to participants of white British ancestry and excluded participants for the following reasons: withdrawn consent; sex mismatch (derived by comparing genetic and reported sex); sex chromosome aneuploidy; poor quality genotyping (missing rate >1.5%); or excess relatedness (>10 putative third degree relatives).

The CARDIoGRAMplusC4D 1000 Genomes based genome wide association study is a meta-analysis of 48 myocardial infarction studies (patients, n=43 676; controls, n=128 199) of people of mainly European descent (about 77%) imputed using the 1000 Genomes phase 1 v3 training set with 38 million variants.^37^ Myocardial infarction was defined based on clinical diagnosis (ICD-9 or ICD-10: international classification of diseases, 9th or 10th revision) and echocardiographic results.^37^ Genetic associations were adjusted for study specific covariates and genomic control.

### Exposure

The exposure was genetically predicted, log transformed serum testosterone (nmol/L).

### Outcomes

We defined thromboembolism (venous thromboembolism, arterial embolism and thrombosis), heart failure, and acute myocardial infarction in the UK Biobank based on self report at baseline or subsequent record linkage to primary diagnosis of hospital episodes and primary cause of death. Algorithmic definitions developed by the UK Biobank cardiac outcome adjudication group were used for myocardial infarction.^43^ We developed classification algorithms for thromboembolism and heart failure using the classifications recommended by the UK Biobank,^44^ as given in supplementary table 1.

### Sensitivity analysis

As a sensitivity analysis, we used genetic variants in the *SHBG* gene region to predict endogenous testosterone. These genetic variants could give conservative estimates because of the pleiotropic effects of sex hormone binding globulin, which might violate the exclusion restriction assumption.^31^ As an additional sensitivity analysis, we repeated the study of sex hormone binding globulin (potentially predicted by variants in the *JMJD1C* and *SHBG* gene regions) obtained from a genome wide association study of 14 938 men and 13 899 women who were mostly of European ancestry.^45^

### Statistical analysis

We obtained associations of genetic variants with the outcomes in the UK Biobank using logistic regression adjusted for the top 10 principal components, age, sex (if relevant), and genotyping array. Inverse variance weighting estimates with fixed effects that account for correlations among genetic variants were used for variants from the *JMJD1C* gene region predicting testosterone and variants from the *SHBG* gene region predicting sex hormone binding globulin. For the variants predicting testosterone from the *SHBG* gene region, we used principal components analysis explaining 99% of the genetic variance of the weighted correlation matrix to stabilise the estimates.^46^

To assess the possibility of bias from violating the exclusion restriction assumption of instrumental variable analysis, we considered variants from each gene region separately because variants from the *SHBG* gene region might operate through sex hormone binding globulin and through testosterone. Because we are using correlated variants from single gene regions, we used the Q statistic^47^ to identify the presence of potential pleiotropy.

We performed analyses using the MendelianRandomisation and TwoSampleMR packages in the R version 3.4.3 software platform (R Development Core Team, Vienna, Austria). Software code in R for implementing the mendelian randomisation analysis, including the principal components analysis, is provided in the supplementary note. We report two sided P**values throughout, with correction for multiple testing to be conservative using a P**value of 0.05/3=0.017, because the three disease outcomes (thromboembolism, heart failure, and myocardial infarction) might not represent the same underlying phenotype. Power calculations were performed using the approximation that the sample size for an instrumental variable analysis compares to that of the same regression analysis with the sample size divided by the r^2^ for the genetic variant on exposure,^48^ using an online tool (https://sb452.shinyapps.io/power/).^49^

### Patient and public involvement

This research was done without public involvement. Participants were not invited to comment on the study design and were not consulted to develop relevant outcomes or interpret the results. Participants were not invited to contribute to the writing or editing of this paper for readability or accuracy.

## Results

We assessed the validity of the genetic variants as instrumental variables in terms of imputation quality, Hardy-Weinberg equilibrium, and associations with potential confounders, and found the following variants fulfilled the inclusion criteria: 234 of 661 variants in the *JMJD1C* gene region predicting endogenous testosterone; one variant in the *JMJD1C* gene region predicting sex hormone binding globulin was not included; 286 of 325 variants in the *SHBG* gene region predicting testosterone; and eight of nine variants in the *SHBG* gene region predicting sex hormone binding globulin. After excluding highly correlated variants, nine variants predicting testosterone from the *JMJD1C* gene region, 21 variants predicting testosterone from the *SHBG* gene region, and seven variants predicting sex hormone binding globulin from the *SHBG* gene region remained. Supplementary figures 1-3 are flow diagrams illustrating the selection of genetic variants. The genetic variants for each analysis are presented in supplementary tables 2-4.

Of 502 642 participants in the UK Biobank, 442 698 (88%) were white British. After we applied sample and genetic quality control, 392 038 participants (179 929 men, 212 109 women) remained, with a mean age of 56.9 years (men 57.2, women 56.7). Of these 392 038 participants, 13 691 had thromboembolism (6208 men, 7483 women), 1688 had heart failure (1186, 502), and 12 882 had myocardial infarction (10 136, 2746). Some participants had comorbidities. The study had 80% power to detect an overall odds ratio of 1.24 per unit change in serum log testosterone for thromboembolism, an odds ratio of 1.65 for heart failure, and an odds ratio of 1.24 for myocardial infarction in the UK Biobank. However, in the CARDIoGRAMplusC4D 1000 Genomes based study, the odds ratio was 1.15 for myocardial infarction (supplementary figure 4).

Table 1 shows that serum testosterone predicted by nine genetic variants from the *JMJD1C* gene region in men from the UK Biobank was positively associated with thromboembolism (odds ratio 2.09, 95% confidence interval 1.27 to 3.46) and heart failure (7.81, 2.56 to 23.81), but not with myocardial infarction (1.17, 0.78 to 1.75). Genetically predicted testosterone was nominally associated with a higher risk of myocardial infarction (1.37, 1.03 to 1.82) in the CARDIoGRAMplusC4D 1000 Genomes based study, with a similar magnitude of estimate as in the UK Biobank (1.11, 0.77 to 1.58), which has fewer cases. The Q statistic only suggested possible heterogeneity for the association with myocardial infarction in the UK Biobank. Generally, we found dose-response relations of testosterone with the outcomes that were consistent with the estimates, as supplementary figure 5 shows. This figure gives the genetic associations with testosterone against the genetic associations with the outcomes, overall and by sex.

**Table 1:** Mendelian randomisation estimates for effect of testosterone (predicted by variants from the *JMJD1C* gene region) on thromboembolism, heart failure, and myocardial infarction

Outcome, data source, and sex of participants	No of cases	Inverse variance weighting			Q statistic	
		Odds ratio (95% CI)	P value		Heterogeneity	P value
**Heart failure**						
UK Biobank						
Men	1186	7.81 (2.56 to 23.81)	0.001*		9.57	0.30
Women	502	0.53 (0.10 to 2.95)	0.47		6.00	0.65
Overall	1688	3.52 (1.38 to 8.95)	0.01*		12.66	0.12
**Thromboembolism**						
UK Biobank						
Men	6208	2.09 (1.27 to 3.46)	0.004*		5.62	0.69
Women	7483	1.49 (0.94 to 2.35)	0.09		1.10	0.99
Overall	13 691	1.74 (1.24 to 2.44)	0.001*		2.18	0.98
**Myocardial infarction**						
UK Biobank						
Men	10 136	1.17 (0.78 to 1.75)	0.44		18.82	0.02
Women	2746	0.91 (0.43 to 1.91)	0.80		1.82	0.99
Overall	12 882	1.11 (0.77 to 1.58)	0.58		13.58	0.09
CARDIoGRAMplusC4D 1000 Genomes based GWAS						
Overall	43 676	1.37 (1.03 to 1.82)	0.03†		4.56	0.80
Both						
Overall	56 558	1.26 (1.01 to 1.57)	0.04†		NA	NA

GWAS=genome wide association study; NA=non-applicable.

Estimates were made by using nine genetic variants from the *JMJD1C* gene region. Odds ratios are per unit increase in log transformed testosterone (nmol/L).

* Association significant after correction for multiple testing (P<0.05/3=0.017).

† Associations at a nominal significance (P<0.05).

Table 2 shows that testosterone predicted by 21 genetic variants from the *SHBG* gene region using principal components analysis was not associated with thromboembolism (odds ratio 1.08, 95% confidence interval 0.83 to 1.42), heart failure (1.78, 0.82 to 3.87), or myocardial infarction (0.79, 0.59 to 1.04). The Q statistic did not indicate heterogeneity. Supplementary table 5 shows that, with inverse variance weighting, sex hormone binding globulin predicted by seven genetic variants from the *SHBG* gene region was positively associated with thromboembolism overall (1.35, 1.02 to 1.79); however, it was inversely associated with myocardial infarction in men (0.69, 0.49 to 0.97) and was not associated with heart failure in the UK Biobank (1.42, 0.64 to 3.16). The Q statistic did not indicate heterogeneity.

**Table 2:** Mendelian randomisation estimates for effect of testosterone (predicted by variants from the *SHBG *gene region) on thromboembolism, heart failure, and myocardial infarction

Outcome, data source, and sex of participants	No of cases	Inverse variance weighting			Q statistic	
		Odds ratio (95% CI)	P value		Heterogeneity	P value
**Heart failure**						
UK Biobank						
Men	1186	2.35 (0.93 to 5.91)	0.07		9.30	0.05
Women	502	0.90 (0.22 to 3.76)	0.90		3.07	0.55
Overall	1688	1.78 (0.82 to 3.87)	0.14		4.41	0.35
**Thromboembolism**						
UK Biobank						
Men	6208	1.07 (0.72 to 1.60)	0.74		3.96	0.41
Women	7483	1.08 (0.75 to 1.56)	0.69		8.28	0.08
Overall	13 691	1.08 (0.83 to 1.42)	0.58		7.61	0.11
**Myocardial infarction**						
UK Biobank						
Men	10 136	0.74 (0.54 to 1.03)	0.07		8.14	0.09
Women	2746	0.95 (0.52 to 1.72)	0.87		4.24	0.37
Overall	12 882	0.79 (0.59 to 1.04)	0.09		6.18	0.19
CARDIoGRAMplusC4D 1000 Genomes based GWAS						
Overall	43 676	0.97 (0.75 to 1.25)	0.84		10.42	0.06
Both						
Overall	56 558	0.88 (0.72 to 1.09)	0.24		NA	NA

GWAS=genome wide association study; NA=non-applicable.

Estimates were made by using 21 genetic variants from the *SHBG* gene region, with principal components analysis to stabilise the estimates. Odds ratios are per unit increase in log transformed testosterone (nmol/L).

## Discussion

### Principal findings

We found that when using variants in the *JMJD1C* gene region, genetically predicted serum testosterone was positively associated with thromboembolism in the UK Biobank. This finding is consistent with the results of a large population based case-control study of venous thromboembolism in the UK^12^ and a small meta-analysis of randomised controlled trials of thromboembolism.^26^ Genetically predicted serum testosterone was also associated with heart failure in men, however, it was not associated with myocardial infarction in the UK Biobank. Genetically predicted serum testosterone was nominally positively associated with myocardial infarction in the larger CARDIoGRAMplusC4D 1000 Genomes based genome wide association study. Sex hormone binding globulin was positively associated with thromboembolism and inversely associated with myocardial infarction, which makes any estimates using variants from the *SHBG* gene region open to pleiotropic effects and difficult to interpret.

### Comparison with other studies

We found no previous mendelian randomisation study that assessed the effect of serum testosterone on thromboembolism or heart failure. One small mendelian randomisation study (n=1454) in men that used rs1799941 from the *SHBG* gene to predict testosterone found no association of testosterone with myocardial infarction.^50^ This finding corresponds with our conclusion that there is no clear association of testosterone predicted by *SHBG* gene region variants with myocardial infarction, and with the results for ischaemic heart disease in our previous mendelian randomisation study.^31^

The effects of testosterone supplementation in men have not been widely studied. This is because in 2003 the Institute of Medicine recommended that large scale trials of TRT should not be conducted until any benefits of testosterone over and above established treatments had been demonstrated in small trials.^51^ Moreover, higher rates of cardiovascular disease in men than in women have previously been attributed to the protective effects of oestrogen in women,^52^ therefore, more investigations have focused on the role of oestrogen rather than that of testosterone in cardiovascular disease. Several large trials examining the effects of oestrogen on cardiovascular disease have been conducted in men and women.^53^ A trial of the cardiovascular disease effects of testosterone in women has been performed,^54^ but such a trial examining testosterone in men has only just started. However, before the exclusive focus on the role of oestrogen as the key sex hormone relevant to cardiovascular disease had been firmly established, testosterone was shown to cause thrombosis in male mice.^55^ Testosterone raises oestrogen in men,^56^ which is a known cause of thromboembolism.^57^ Testosterone also increases platelet aggregation through thromboxane A2,^58^ ^59^ which could underlie any effects on thromboembolism. In mice, testosterone induces cardiac myocyte hypertrophy and antiandrogens improve cardiac function and reduce mortality.^60^ Testosterone also raises endothelin, which causes ischaemic heart disease.^61^ We found an association between endogenous testosterone and a higher risk of thromboembolism, heart failure, and myocardial infarction, particularly in men. These results extend and complement previous findings of an association between endogenous testosterone and a higher risk of ischaemic heart disease and ischaemic stroke, particularly in men.^31^

Taken together, these findings suggest a common factor could underlie thromboembolism, heart failure, and myocardial infarction, and explain higher rates of these conditions in men than in women. Several effective treatments for cardiovascular disease including statins, digoxin, and some antihypertensives, such as spironolactone, reduce endogenous testosterone.^20^ ^62^ Whether testosterone contributes to the mechanism of action of these treatments is not known, however the established targets of many other treatments for ischaemic heart disease do not seem to have clear genetic validation.^63^

### Strengths and limitations

We used a new method to obtain unconfounded estimates^30^ of the effects of endogenous testosterone on thromboembolism, heart failure, and myocardial infarction. We used a validated myocardial infarction classification algorithm^43^ and validated the genetic variants as instrumental variables in the UK Biobank. Several statistical techniques and a validation study for myocardial infarction were also applied. However, our study had several limitations.

Mendelian randomisation has stringent assumptions.^29^ We checked for potential confounders of the genetic variant and disease outcome associations. We also used a Q statistic to test statistically for pleiotropic effects that might indicate violations of the exclusion restriction assumption.^47^ In addition, the genetic predictors of serum testosterone are not independent of sex hormone binding globulin because all the autosomal gene regions associated with testosterone concentrations at genome wide significance levels^38^ ^64^ are also associated with sex hormone binding globulin.^45^ Therefore, variants predicting testosterone from the *SHBG* gene region are open to the pleiotropic effects of sex hormone binding globulin. However, the estimates from *JMJD1C* variants could be least biased by sex hormone binding globulin because *JMJD1C* is probably relevant to male fertility^65^ ^66^ and might have functional relevance to testosterone. Compared with estimates from the *JMJD1C* gene region, the observed reverse estimates from the *SHBG* gene region are consistent with the antagonistic relation between the bioavailability of testosterone and sex hormone binding globulin. Furthermore, we used genetic predictors of serum testosterone derived from a sample of men to estimate testosterone in women, therefore the estimates for women should be interpreted with caution. However, because levels of testosterone are higher in men than in women, the stronger associations we found in men than in women are consistent with testosterone as the causal mechanism.

Although the largest available sources of genetic associations with thromboembolism and heart failure were used, the relatively low number of participants with heart failure led to imprecise estimates and wide 95% confidence intervals. The response rate of around 5.5% for the UK Biobank resulted in the recruitment of generally healthier participants,^67^ which might have biased towards the null. This could explain the discrepancy between the estimates from the UK Biobank and the myocardial infarction case-control study, although the difference might also have been because of a lack of power in the UK Biobank. Therefore, our positive estimates for thromboembolism and heart failure could be underestimated because of survivor bias in the UK Biobank. 

Endogenous testosterone decreases with poor health, and the testosterone genome wide association study did not adjust for health status. Therefore, the estimates for genetic variants on testosterone in the genome wide association study could be biased towards the null and might be imprecise; however, such bias is probably minimal.^68^ Genetic associations with testosterone were estimated in men aged 50-75. We assume that these genetic associations reflect differences in testosterone concentrations that are also present across the age range (40-69) of UK Biobank participants. In addition, the UK Biobank has implemented high quality procedures to capture and classify events, but complete diagnostic accuracy is impossible. However, misclassification is unlikely to be related to genetic makeup, so any random misclassification of the outcomes would probably bias towards the null. 

Our estimates represent average causal effects across the population and so might not be relevant for all subgroups of the population. We also considered venous and arterial thromboembolism together because they might share common risk factors,^69^ however, considering venous and arterial thromboembolism separately gives a similar interpretation (supplementary table 6). Finally, our study compared groups with genetically predicted higher and lower levels of endogenous testosterone to make inferences about the expected effect of raising testosterone. However, there are several qualitative differences between the comparisons that could limit the applicability of our findings to assess the effect of increasing testosterone levels. Specifically, genetic variants are associated with small but lifelong changes in endogenous testosterone levels that occur from modulating a particular biological pathway, whereas testosterone supplementation typically occurs later in life and increases testosterone levels by a relatively large amount.

### Clinical implications

From a clinical perspective, our study suggests that lifelong endogenous testosterone could have a role in thromboembolism, heart failure, and possibly myocardial infarction, particularly among men. These findings provide another strand of evidence consistent with the cardiovascular warnings about TRT issued by regulators. Further evidence is needed to clarify whether our findings are relevant to the higher rates of these diseases in men than in women,^70^ ^71^ or suggest that agents that lower testosterone would be protective. Additional research is also required comparing the effects of endogenous testosterone with those of exogenous testosterone.

### Conclusions

Our study suggests that endogenous testosterone could have a role in thromboembolism, heart failure, and myocardial infarction in men. It might be worth considering whether existing treatments that modulate endogenous testosterone could be used for these conditions.

**What is already known on this topic**

Testosterone replacement therapy has increased globally and evidence of its role in cardiovascular disease is unclear
A recent mendelian randomisation study showed some evidence indicating that genetically predicted endogenous testosterone is positively associated with ischaemic heart disease and ischaemic stroke, especially in men
Understanding the causal role of testosterone in other types of cardiovascular disease is important for public health and regulators

**What this study adds**

This study suggests that genetically predicted endogenous testosterone using variants in the *JMJD1C* gene region is detrimental for thromboembolism, heart failure, and myocardial infarction, especially in men
Sex hormone related mechanisms have a role in these cardiovascular diseases

## Notes

Ethical approval: The UK Biobank received ethical approval from the research ethics committee (REC reference for UK Biobank 11/NW/0382) and participants provided written informed consent. No ethics approval was acquired for the analysis using publicly available data (CARDIoGRAMplusC4D 1000 Genomes based genome wide association study).

Data sharing: The data reported in this paper are available by application directly to the UK Biobank. The genetic associations with the outcomes in the UK Biobank and CARDIoGRAMplusC4D consortium are provided in the supplementary data. Software code in R for implementing the mendelian randomisation analysis, including the principal components analysis, is provided in the supplementary note.

The lead author (the manuscript’s guarantor) affirms that this manuscript is an honest, accurate, and transparent account of the study being reported; that no important aspects of the study have been omitted; and that any discrepancies from the study as originally planned (and, if relevant, registered) have been explained.
